# Quantification and phenotypic characterisation of peripheral IFN-γ producing leucocytes in chickens vaccinated against Newcastle disease

**DOI:** 10.1016/j.vetimm.2017.10.001

**Published:** 2017-12

**Authors:** S.H. Andersen, L. Vervelde, K. Sutton, L.R. Norup, E. Wattrang, H.R. Juul-Madsen, T.S. Dalgaard

**Affiliations:** a Department of Animal Science, Aarhus University, Tjele, Denmark; b The Roslin Institute and Royal (Dick) School of Veterinary Sciences, University of Edinburgh, Easter Bush, Midlothian, UK; c Department of Virology, Immunobiology and Parasitology, National Veterinary Institute, Uppsala, Sweden

**Keywords:** Chicken, Vaccines, Intracellular cytokine staining, Interferon-γ, Flow cytometry, T cells

## Abstract

The aim of this study was to optimise and evaluate an intracellular cytokine staining (ICS) assay for assessment of T cell IFN-γ responses in chickens vaccinated against Newcastle disease (ND). We aimed to validate currently available antibodies to chicken IFN-γ using transfected CHO cells. Moreover, this ICS assay was evaluated for use to detect mitogen and antigen induced IFN-γ production in chicken peripheral blood leucocytes.

Chickens from an inbred white leghorn line containing two MHC haplotypes, B19 and B21, were divided into three experimental groups; one group was kept as naive controls, one group was vaccinated intramuscularly twice with a commercial inactivated ND virus (NDV) vaccine, and the last group was vaccinated orally twice with a commercial live attenuated NDV vaccine. PBMC were *ex vivo* stimulated with ConA or with NDV antigen. The ICS assay was used to determine the phenotype and frequency of IFN-γ positive cells. ConA stimulation induced extensive IFN-γ production in both CD3^+^TCRγδ^+^ (γδ T cells) cells and CD3^+^TCRγδ^−^ cells (αβ T cells), but no significant differences were observed between the experimental groups. Furthermore, a large proportion of the IFN-γ producing cells were CD3^−^ indicating that other cells than classic T cells, secreted this cytokine. NDV antigen stimulation induced IFN-γ production but to a lower extent than ConA and with a large variation between individuals. The CD3^+^TCR1γδ^−^CD8α^+^ (CTL) population produced the highest NDV specific IFN-γ responses, with significantly elevated levels of IFN-γ producing cells in the B19 chickens vaccinated orally with live attenuated NDV vaccine. This was not the case in the B21 animals, indicating a haplotype restricted variation. In contrast, the CD3^+^TCR1γδ^−^CD4^+^ (Th) population did not show a significant increase in IFN-γ production in NDV stimulated samples which was in part due to a high number of IFN-γ producing cells after incubation with medium alone. In conclusion, an ICS assay for phenotyping of IFN-γ producing chicken leukocytes was set up that proved useful in identifying cytokine producing cells upon either mitogen or antigen-specific stimulation.

## Highlights

• An avian ICS assay for detection of chIFN-γ was established.
• Commercially available chIFN-γ antibodies were evaluated using tranfected CHO cells.
• Functional T cell responses were addressed in NDV vaccination study.
• Circulating T cells producing IFN-γ were quantified and phenotyped by flow cytometry.

## 1 Introduction

Several T cell-mediated immune mechanisms are essential for the control of viral infections and play key roles in vaccine-induced antiviral immunity. Thus, accurate quantification of specific T cell responses is a focus of interest to understand protective immunity and to investigate vaccine efficacy. Studies of antigen-specific T cells may comprise functional analyses *ex vivo* after activating the cells with recall antigen. The lymphocyte activation can then be evaluated by different methods using functional read-outs, such as proliferation, expression of surface activation markers, or cytokine production (Thiel et al., 2004). To detect cytokine producing cells the intracellular cytokine staining (ICS) technique is a particularly useful method, which simultaneously allows visualisation of single cells, their cytokine production, frequency and phenotype. This method is based on antigen-activation of leukocyte cultures in the presence of a secretion inhibitor prior to combined surface and intracellular staining followed by flow cytometry analysis (Suni et al., 1998).

ICS has been extensively used in human medical research, to address antigen-specific T cell responses in settings such as experimental vaccination. Quantification of the number of T cells, which produce the effector cytokine, IFN-γ, in response to recall stimulation, has been a particularly popular method for years. However, it has been shown that vaccination in humans generates a broad and complex T cell cytokine response (De Rosa et al., 2004). Hence, proper evaluation of the response will therefore require coordinate measurements of several cytokines, which makes ICS and polychromatic flow cytometry invaluable tools. The ICS method is not yet widely used in avian immunology research and only a few published reports exist (Ariaans et al., 2008, Huang et al., 2011, Ruiz-Hernandez et al., 2015), an example being a methodological study describing ICS applied to study IFN-γ production in polyclonal stimulated splenocytes (Ariaans et al., 2008). Furthermore, only a few antibodies specific for chicken IFN-γ (chIFN-γ) are currently commercially available (Table 1). In other domestic species such as pigs and cattle, the ICS method has been used with success in differentiating cells based on phenotypic and cytokine profile following polyclonal stimulation and antigen specific stimulation (Sassu et al., 2017, Sopp and Howard, 2001). The purpose of the present study was therefore to further evaluate, optimise and develop protocols for analyses of the chicken cellular response of not only polyclonal stimulation but also antigen-specific stimulation of Newcastle disease virus (NDV) specific T cells.

**Table 1:** Chicken IFN-γ antibodies tested in intracellular cytokine staining assay.

Antibody ID	Isotype	Reference	Commercial availability
5C.123.08	Mouse monoclonal IgG1	Lambrecht et al. (2000)	IFN-γ CytoSet™ ELISA, Invitrogen
5C.123.02 (biotin)	Mouse monoclonal IgG1	Lambrecht et al. (2000)	IFN-γ CytoSet™ ELISA, Invitrogen
Rabbit anti Chicken Interferon-γ	Purified polyclonal rabbit IgG	Weining et al. (1996)	BioRad
Mab80	Mouse monoclonal IgG1	Ariaans et al. (2008)	None

NDV is an avian paramyxovirus that causes Newcastle disease (ND), a highly contagious disease which represents a severe economic problem for the poultry industry (Alexander, 2001). In the present study, NDV specific immune responses were induced in chickens using a vaccination model. Live attenuated (LA) and inactivated (IA) NDV vaccines are commercially available and while both types of vaccine formulations induce antibody responses LA NDV vaccines are considered better inducers of cellular immunity (Al-Garib et al., 2003, Zoth et al., 2008, Lambrecht et al., 2004, Rauw et al., 2009, Jayawardane and Spradbrow, 1995). A number of studies in poultry have shown that IFN-γ produced by T cells after *ex vivo* stimulation correlate with cellular immunity after vaccination or infection (Breed et al., 1997, Karaca et al., 1996, Martin et al., 1994, Prowse and Pallister, 1989). NDV-specific cell-mediated immunity induced by live vaccines was earlier demonstrated in peripheral blood and spleen by *ex vivo* recall stimulation and assessment of chicken IFN-γ production by capture ELISA and ELISPOT (Ariaans et al., 2008, Lambrecht et al., 2004, Rauw et al., 2009, Rauw et al., 2010). In the present work, we optimised and evaluated an ICS method for assessment of specific T cell responses in peripheral blood from NDV vaccinated chickens by quantification and phenotypic characterisation of IFN-γ producing cells.

## 2 Materials and methods

### 2.1 Method establishment and optimisation

#### 2.1.1 Transfection of CHO cells and test of antibodies for intracellular IFN-γ staining

To evaluate antibodies for assessment of intracellular IFN-γ, Chinese Hamster Ovary (CHO) cells were transfected with full length chIFN-γ cDNA (Lawson et al., 2001) or empty pCI-neo vector. CHO cells were maintained in Ham’s F-12 Nutrient Mixture (F-12) with Glutamax (Gibco/Thermo Fischer Scientific, Waltham, MA, USA) supplemented with 10% FCS until 90% confluency before transfection. CHO cells were transfected with Lipofectamine 2000™ (Invitrogen, Carlsbad, CA, USA) according to the manufacturer’s instructions. After at least 3 weeks the selected CHO cells were sub-cultivated into 6 well plates in medium with or without Brefeldin A (BFA) (Sigma-Aldrich) and left overnight at 41 °C, 5% CO_2._ Subsequently the cells were trypsinised, washed in PBS and used for intracellular IFN-γ staining using the BD Cytofix/Cytoperm™ Kit (BD Biosciences, San Jose, CA, USA) according to the manufacturer’s instructions. After staining, cells were re-suspended in BD Perm/Wash buffer and used for flow cytometry analyses.

Antibodies for assessment of IFN-γ expression in CHO cells included 5C.123.08 and 5C.123.02 from the ELISA kit Chicken IFN-γ CytoSet™ (Invitrogen). 5C123.08 was used in combination with Goat anti-Mouse IgG1 Secondary Antibody, Alexa Fluor^®^ 647 (Invitrogen) and 5C.123.02 was labelled with APC using the LYNX Rapid APC Antibody Conjugation Kit^®^ (BioRad, Oxford, UK), according to the manufacturer’s instructions. Furthermore we tested Mab80 commercially conjugated with APC (Ariaans et al., 2008) as well as rabbit polyclonal chIFN-γ IgG antibody (BioRad) in combination with secondary goat anti rabbit IgG FITC (Beckman Coulter, Brea, CA, cat no 732745) (Table 1).

#### 2.1.2 Optimisation of PBMC surface staining conditions

Blood, spleen and bone marrow were obtained from 3 to 9-week-old animals *post-mortem* and cells were purified by Ficoll density gradient as described previously (Dalgaard et al., 2010b, Sutton et al., 2015). Bone marrow-derived dendritic cells (BMDC) cultures were generated as described (Wu et al., 2010). Cells were harvested with TrypLX (Thermo Scientific, CA. U.S.A). Cell concentrations were adjusted to 1 × 10^7^cells/ml and stained in 96-U bottom plates at 100 μl/well with mouse anti-chicken CD3 (CT3), Bu1-Alexa Fluor^®^ 647 (AV20), KUL01 (Southern Biotech, USA), isotype controls, mouse IgG1 (NCG01, Thermo Scientific) and mouse IgG1-Alexa Fluor^®^ 647 (Thermo Scientific). To test the effect of sodium azide and the temperature of the FACS buffer we used PBS (pH 7.4) or PBS supplemented with 0.05% sodium azide (PBS/azide) for 30 min on ice or room temperature (RT). Cells stained with unconjugated antibodies were incubated with goat anti-mouse IgG1 Alexa Fluor^®^ 647 (Southern Biotech, USA), for 30 min on ice or RT. Live cells were analysed by Sytox Blue exclusion.

#### 2.1.3 Optimisation of PBMC stimulation and intracellular IFN-γ staining

Peripheral blood from inbred L21 chickens (4 unvaccinated and 4 NDV vaccinated) was collected in BD Vacutainer^®^ Blood Collection Plasma Tube coated with 60 USP Units of Sodium Heparin (BD, Franklin Lakes, NJ, USA). PBMC were purified from peripheral blood by Ficoll density gradient centrifugation as described earlier (Dalgaard et al., 2010b). Isolated PBMC were re-suspended at a concentration of 1 × 10^7^ cells/ml in different media to test optimal culture conditions for *ex vivo* stimulation. The culture media tested were serum free X-VIVO 15 medium containing 2 mM l-glutamine (Cambrex/Lonza, Walkersville, MD), serum free CTL-test medium™ (Cellular Technology Ltd., Bonn, Germany) and R10 medium (RPMI containing 2 mmol/l-Glutamine (Cambrex/Lonza, Walkersville, MD) supplemented with 10% FCS (Cambrex/Lonza)). All culture media were supplemented with 100 U/ml penicillin and 100 μg/ml streptomycin (Cambrex/Lonza), and subsequently transferred to 96-well plates at 100 μl/well (1 × 10^^6^cells/well).

NDV antigen was prepared from NDV-vaccine which was inactivated by UV light as earlier described (Dalgaard et al., 2010a) with a few modifications. In brief, Poulvac NDW vaccine (10^6^–10^6.6^ EID50 per dose, Fort Dodge Animal Health Ltd., Southampton, UK) was re-suspended in various culture media and UV-inactivated using a UV cross-linker (UVC500, Hoefer, San Francisco, CA). The solution was split into two tubes and one half of the antigen preparation was in addition to the UV inactivation also treated with ultrasound using a Vibra cell™ VC130 (Sonics and Materials Inc). Both antigen preparations were mixed 1:1 (v/v) before aliquoted and stored at −20 °C until use.

Different concentrations of ConA (Sigma-Aldrich) and NDV antigen were used for stimulation of PBMC from the individual chickens in combination with BFA in a total volume of 200 μl/well. Cells were incubated at 41 °C, 5% CO_2_ for various lengths of time and subsequently stained for IFN-γ using the BD Cytofix/Cytoperm™ Kit (BD Biosciences) according to the manufacturer’s instructions. In brief, 100 μl BD Cytofix/Cytoperm solution was added and cells were incubated for 20 min (at 4 °C as recommended by the manufacturer). Subsequently, the cells were washed twice with BD Perm/Wash buffer for 5 min at 600 × *g*, followed by staining with rabbit polyclonal anti-chIFN-γ IgG antibody (BioRad) and secondary goat anti-rabbit IgG FITC (Beckman Coulter, Brea, CA, cat no 732745). To test for unspecific intracellular staining of primary and secondary antibodies, additional controls were included: Rabbit anti chicken IgG FITC (Sigma cat no 8320-02) as well as secondary goat anti rabbit IgG FITC (Beckman Coulter, cat no 732745) After staining, cells were re-suspended in BD Perm/Wash buffer and immediately used for flow cytometry analyses.

### 2.2 NDV vaccination experiment

#### 2.2.1 Animals and experimental design

Chickens were used from an inbred white leghorn line L21, AU/DIAS lines (Miller et al., 2004), containing two MHC haplotypes B19 and B21 (Dalgaard et al., 2009). Offspring used in the experiment were produced from NDV-vaccinated parents. Hence, experimental immunisations were initiated when the chickens were 4 weeks of age to avoid influence from maternal antibodies. In total 24 B19 chickens and 24 B21 chickens were divided into 3 experimental groups. One group was kept as naive controls without mock vaccination, one group was vaccinated intramuscularly with a commercial inactivated NDV vaccine (Poulvac iND Vet, Fort Dodge) at 4 and 7 weeks of age, and the third group was vaccinated orally with a commercial live attenuated NDV vaccine (ND C2, Intervet) at 4 and 7 weeks of age. Peripheral blood samples were collected for serum analyses throughout the experiment at weeks 0, 1, 3, 4, 8, 11, 13, post-primary vaccination (PV1) without sacrificing the chickens. Additional blood was drawn for the ICS assay from the jugular vein at weeks 8 and 13 which corresponds to weeks 5 and 10 post-secondary vaccination (PV2), in BD Vacutainer^®^ Blood Collection Plasma TubeHeparin.

#### 2.2.2 MHC haplotyping

The offspring used in the experiment were produced from homozygous MHC-characterised parents and their MHC haplotypes were confirmed by genotyping of the LEI0258 microsatellite locus (Fulton et al., 2006, McConnell et al., 1999) by PCR-based fragment analysis and gel documentation (Dalgaard et al., 2005). Genomic DNA was isolated from peripheral blood using the ArchivePure™DNA Blood Kit (5 PRIME GmbH, Hamburg, and Germany) according to the manufacturer’s instructions.

#### 2.2.3 ELISA for NDV specific antibodies in serum

The Proflock Plus NDV ELISA kit (Synbiotics, San Diego, CA) was used to measure NDV-specific IgY (H + L) levels in serum. The ELISA was performed according to the manufacturer’s instructions. Briefly, 96-well microtiter plates, coated with NDV antigen, were incubated with serum samples (diluted 1:100) and controls included in the kit. The result was recorded as optical density (OD) at 405 nm, and the antibody titre was calculated from a sample to positive ratio (SP) as LOG10titer = (1.464 × LOG10 SP) + 3.74, SP being ((sample absorbance) − (average normal control absorbance))/(average positive control absorbance − average negative control absorbance).

#### 2.2.4 PBMC stimulation

PBMC from heparinised blood were isolated and re-suspended at a concentration of 1 × 10^7^ cells/ml in serum free X-VIVO 15 medium supplemented with 100 U/ml penicillin and 100 μg/ml streptomycin. The cells were subsequently transferred to 96-well plates at a volume of 100 μl/well (1 × 10^6^ cells/well).

PBMC were stimulated with 10 ug/ml of ConA in a total volume of 200 μl/well. BFA was added in a final concentration of 5 μg/ml to all cultures including medium controls. Both ConA and BFA were added to the cell cultures at the start of the incubation at 41 °C, 5% CO_2_ in a humid atmosphere for 16 h. ConA stimulation was performed on PBMC from the experimental chickens at 5 weeks PV2. In contrast to stimulation with ConA, for the NDV antigen stimulation, PBMC from experimental chickens were left to rest overnight at 41 °C, 5% CO_2_ before the NDV antigen was added at a concentration of 1 vaccine doses in 20 μl per well. To ensure antigen presentation, cells were incubated for a further 6 h before the addition of BFA (final conc. 5 ug/ml) to all cells. Cells were subsequently incubated for a further 16 h before staining and flow cytometry analysis. Stimulation with NDV antigen was performed on PBMC from the experimental chickens at 10 weeks PV2 only.

#### 2.2.5 Staining antibodies used in ICS assay

All monoclonal antibodies for phenotypic markers were obtained from Southern Biotech (Birmingham, AL, USA) except the rabbit polyclonal anti-chIFN-γ antibody, which was purchased unlabelled and FITC conjugated using the LYNX Rapid Fluorescein Conjugation Kit^®^ (BioRad) following the manufacturer’s instructions. Two different surface staining panels were used in the flow cytometry analyses of samples from the animal experiment. **Panel 1** (ConA-stimulated samples): IFN-γ-FITC, CD3-SPRD (PE-Cy5), TCRγδ-biotin (TCR-1), Strepavidin-PE-Cy7. **Panel 2** (NDV-stimulated samples) IFN-γ-FITC, CD4-RPE, CD3-SPRD, CD8α-Cy5, TCRγδ-biotin (TCR-1), Strepavidin-PE-Cy7. In both panels the fixable LIVE/DEAD^®^ near-IR fluorescent amine reactive dye (Invitrogen-Molecular Probes, Eugene, OR, USA) was included as viability dye. Titration of all antibodies was done prior to the experiment in order to determine optimal staining concentrations. For both panels, negative fluorescence minus one (FMO) controls (Roederer, 2001) were included to determine the level of background fluorescence in each channel to ensure correct gating.

#### 2.2.6 Surface staining of PBMC

Prior to intracellular staining and flow cytometry analysis, cells were stained for selected surface markers. After incubation with ConA or antigen, cells were treated with EDTA (2 mM) for 10 min at 41 °C, 5% CO_2._ Cells were harvested by gentle pipetting and pelleted at 600 × *g* for 5 min. Cells were stained for surface markers and viability dye in a total volume of 100 μl PBS for 20 min at RT in the dark. Washing was carried out in 150 μl of PBS at 600 × *g* for 5 min. Subsequently, PE-Cy7-conjugated streptavidin was added in a total volume of 100 μl PBS and cells were incubated for 20 min at RT followed by two washes with PBS. Staining was performed in PBS without azide based on the results show in Supplementary Fig. 1 and the manufacturer’s recommendations for fixable LIVE/DEAD^®^ near-IR fluorescent amine reactive dye.

#### 2.2.7 Intracellular cytokine staining

After the surface staining, fixation and permeabilisation was carried out using the BD Cytofix/Cytoperm™ Kit (BD Biosciences) according to the manufacturer’s instructions as described above. The PBMC were stained with FITC-conjugated rabbit polyclonal anti-chIFN-γ IgG antibody (BioRad) in a total volume of 100 μl BD Perm/Wash buffer for 20–30 min. Finally, cells were washed twice and re-suspended in PBS with 1% paraformaldehyde (Electron Microscopy Sciences, Hatfield, PA, USA). For the ConA-stimulated samples, at least 10,000 live cells were acquired, and for Ag-stimulated samples, at least 1000–2000 live CD3^+^ cells were acquired. If the viability in medium controls were below 70% all samples from that individual were excluded from statistical analysis.

#### 2.2.8 Flow cytometry

All flow cytometry analyses were performed on a BD FACSCanto™ or a BD Fortessa™ (BD Biosciences) equipped with a 488 nm blue laser and a 633 nm red laser. Acquisition and analysis were done using the FACSDiva software version 5.0.3 (BD Biosciences) and FlowJo version 10 (TreeStar Inc, Ashland, OR, USA).

#### 2.2.9 Ethics and animal care

Animals used for optimising flow cytometry surface staining were housed in premises licensed under a UK Home Office Establishment License within the terms of the UK Home Office Animals (Scientific Procedures) Act 1986. Housing and husbandry complied with the Code of Practice for Housing and Care of Animals Bred, Supplied or Used for Scientific Purposes and were overseen by the Roslin Institute Animal Welfare and Ethical Review Board. Animals were culled by schedule one methods authorized by Animals (Scientific Procedures) Act 1986.

The animals for PBMC ICS optimisation as well as the NDV vaccination experiment were kept according to the protocols approved by the Danish Animal Experiments Inspectorate and complied with the Danish Ministry of Justice Law no. 382 (10th June 1987) and Acts 739 (6th December 1988) and 333 (19th May 1990) concerning animal experimentation and care of experimental animals. The license to conduct the animal experiment was obtained by Dr. Juul-Madsen. The experiment was conducted according to the ethical guidelines.

#### 2.2.10 Statistical analysis

All data are presented as mean values with 95% confidence intervals and values with non-overlapping confidence intervals indicate significant differences. An unpaired T test was used to compare the chIFN-γ response in the different MHC and treatment groups.

## 3 Results

### 3.1 CHO cell ICS staining

**Fig. 1:**
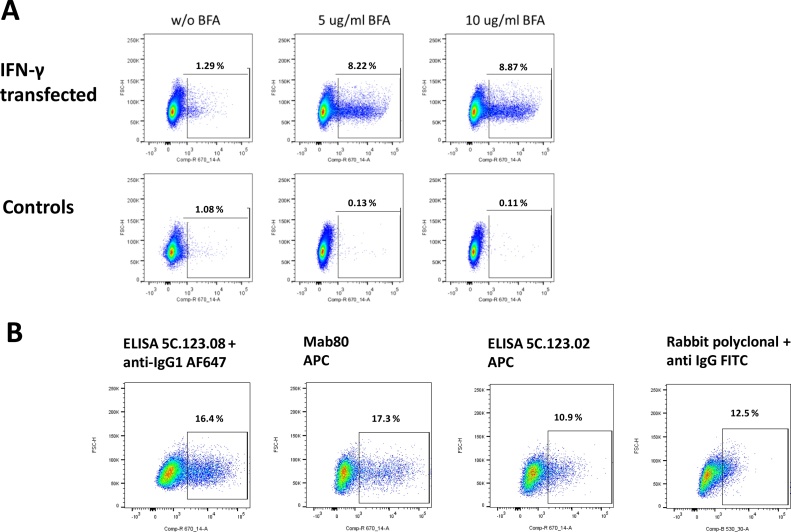
**Intracellular staining of IFN-γ in transfected CHO cells.** A) CHO cells were either transfected with plasmids containing the chicken IFN-γ gene or empty plasmids (mock controls). Cells (day 22 after transfection) were cultured with or without BFA for 18 hours and subsequently stained intracellularly using monoclonal anti-chicken IFN-γ antibody (Mab80) conjugated with APC. Representative samples are show with frequency of IFN-γ+ cells indicated above gate. B) Comparison of transfected CHO cells (day 30 after transfection) cultured with 10 μg/ml BFA for 18 hours and subsequently stained intracellularly with either commercial ELISA capture antibody (5C.123.08) & A647 conjugated secondary anti-mouse IgG1 or monoclonal anti-chicken IFN-γ antibody directly conjugated with APC (Mab80) or commercial ELISA detection antibody (5C.123.02) directly conjugated with APC or polyclonal rabbit anti-chicken IFN-γ antibody & FITC-conjugated secondary goat anti-rabbit IgG. Representative samples are show with frequency of IFN-γ+ cells indicated above gate.

Transfected cells expressing a cytokine of interest are useful for optimisation and standardisation of intracellular cytokine staining protocols. We used CHO cells transfected with the pCI-neo vector containing *chIFN-γ* to test commercially available anti-chIFN-γ antibodies. It was possible to detect chIFN-γ in some resting transfected CHO cells but the frequency of chIFN-γ producers increased if cells were treated with BFA (Fig. 1A). All four tested antibodies to chIFN-γ detected recombinant chIFN-γ produced by CHO cells (Fig. 1B). The three monoclonal antibodies as well as the polyclonal serum, stained comparable frequencies of chIFN-g producing cells but the intensity of the staining (MFI) was generally low for the polyclonal rabbit antibody. In contrast, the lower MFI was not an issue when staining native chIFN-γ in PBMC indicating better affinity for the native protein rather than the recombinant (Fig. 3).

### 3.2 Optimisation of PBMC surface staining conditions

Surface staining of chicken leucocytes for flow cytometry may be carried out at 4°C or at RT. Different laboratories have different procedures for staining cells for FACS analysis and the rationale for incubating cells on ice/4°C is to avoid unspecific staining either caused by cells endocytosing the staining antibodies or binding *via* Fc receptors. Hence, we sought to investigate the ability of avian phagocytes and lymphocytes to endocytose or bind mouse IgG aspecifically, and whether staining conditions affected staining frequencies by flow cytometry. Using highly phagocytic cells, *i.e.* BMDC, and a mixture of various cell populations, *i.e.* PBMC, cell staining on ice or at RT and in the presence of PBS or PBS/azide was analysed by flow cytometry (Supplementary Fig. 1). For PBMC (Fig. S1A) or BMDC (Fig. S1B) stained under the various conditions neither the reference (B, T and phagocytic cells) nor the mouse isotype IgG staining frequencies were affected significantly by buffer composition or temperature conditions. Cell staining with PBS alone gave higher cell percentages compared to PBS/azide potentially indicating unspecific staining and using a conjugated antibody gave consistent cell frequencies between the different buffers compared to indirect cell staining. When we compared the staining of highly phagocytic cells, BMDC, with unconjugated and conjugated isotype controls there was little differences in their binding capacity indicating that the unconjugated antibody is not endocytosed before addition of the secondary antibody. The slight increase in unconjugated mouse IgG binding may possibly be due to the unspecific binding of the secondary antibody. In conclusion, staining avian lymphocytes at RT with PBS did not significantly alter surface staining or enhance the uptake or binding of mouse IgG and was therefore used in further analysis.

### 3.3 Optimisation of PBMC stimulation and intracellular IFN-γ staining

During optimisation of the PBMC protocol several variables were addressed (conclusions summarised in Supplementary Table 1, data not shown). It was observed that an overnight rest of the PBMC before stimulation with viral antigen increased the observed frequencies of IFN-γ producing cells. This was in contrast to polyclonal stimulation with ConA where immediate stimulation after PBMC isolation gave the highest frequencies of IFN-γ producing cells. Different culture medium types were tested and stimulation of PBMC from naive chickens with ConA (10 μg/ml) showed slightly higher (but not significant) frequencies of IFN-γ producing cells in serum free medium (X-VIVO15 or CTL) as compared to R10 medium. After overnight stimulation with 10 μg/ml of ConA in the presence of 5 μg/ml of BFA, it was shown that if cells were harvested with 2 mM of EDTA, increased frequencies of IFN-γ producing cells were detected. As expected, stimulation with 10 μg/ml of ConA, in the presence of 10 μg/ml of BFA for 6 h, produced a lower IFN-γ response as compared to the overnight ConA stimulation and BFA treatment. To test for unspecific intracellular staining of primary and secondary antibodies, additional controls were included. PBMC staining was performed using an irrelevant rabbit polyclonal antibody (Rabbit anti chicken IgG FITC) or secondary goat anti rabbit IgG FITC without primary antibody (Supplementary Fig. 2). No unspecific staining was observed, however some CD8^−^ cells stained positive as expected, probably due to IgG bound to the surface of B-cells.

### 3.4 Antibody responses to NDV vaccination

**Fig. 2:**
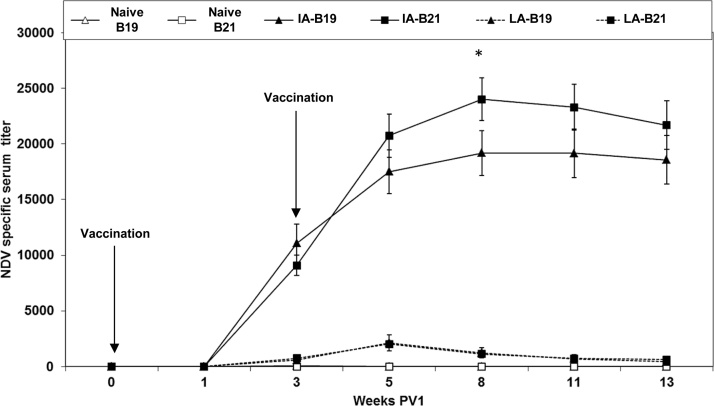
**NDV-specific antibodies in serum measured by ELISA.** Chickens of two different MHC haplotypes B19 and B21 were used for the vaccination experiment. One group was left as naive controls (naive), one group was intramuscularly vaccinated with commercial inactivated vaccine (IA) twice (4 and 7 weeks of age) and the last group was orally vaccinated with commercial live attenuated vaccine (LA) twice (4 and 7 weeks of age). Vaccination times are indicated as week 0 PV1 and week 3 PV1. Each value represents a mean of 8 individual determinations ± 95% confidence intervals. Significant differences between MHC haplotypes within the same treatment group are indicated by * (P < 0.05).

To monitor antibody responses to the NDV vaccinations in the experimental chickens NDV-specific antibody titres in serum were measured by ELISA (Fig. 2). All vaccinated chickens seroconverted to NDV in response to the vaccination. The chickens immunised i.m. with the inactivated (IA) vaccine showed significantly higher serum titres than chickens immunised with the live attenuated (LA) vaccine. No MHC-related differences were seen in the group vaccinated with the live attenuated vaccine. In contrast, when using the inactivated vaccine, B21 chickens showed significantly higher titres 8 weeks PV1 (corresponding to 5 weeks PV2) after which titres declined to the same levels as those of the B19 chickens. Thus, all vaccinated chickens had mounted NDV-specific immune response at the sampling time-points for IFN-γ stimulation experiments.

### 3.5 IFN-γ production in PBMC stimulated *ex vivo* with ConA

**Fig. 3:**
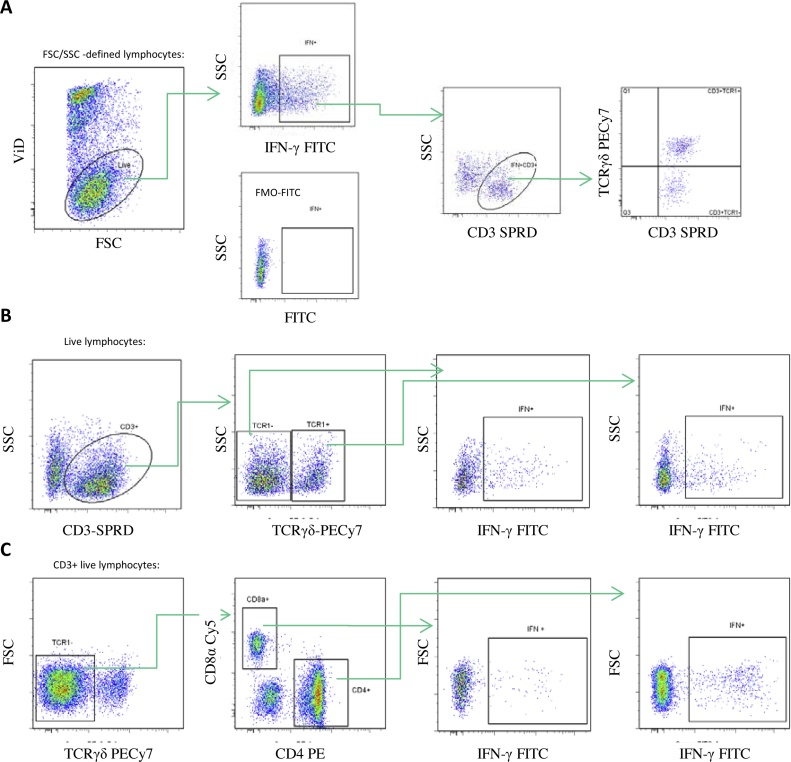
**Gating strategies for flow cytometry analyses.** A) The gating strategy used for Fig. 4A (staining panel 1) and Fig 5A (staining panel 2) was: FSC/SSC defined lymphocytes (not shown), live lymphocytes by viability dye (ViD) exclusion using Near-IR live/dead cells stain, IFNγ^+^, IFNγ ^+^CD3^+^ and TCRγδ^+^/TCRγδ^-^. For Fig. 5A the IFNγ^+^CD3^+^TCRγδ^-^ cells were further divided into CD4^+^ and CD8α^+^ cells (not shown). Below the FMO-FITC negative control is shown. B) The gating strategy for Fig. 4B and 4C (staining panel 1) was: FSC/SSC defined lymphocytes (not shown), live lymphocytes by ViD exclusion (not shown), CD3^+^, TCRγδ^+^/TCRγδ^-^, IFNγ^+^. C) The gating strategy for Fig. 5B and 5C (staining panel 2) was: FSC/SSC defined lymphocytes (not shown), live lymphocytes by ViD exclusion (not shown), CD3^+^ (not shown), TCRγδ^-^, CD4^+^/CD8α^+^, IFNγ^+^.

**Fig. 4:**
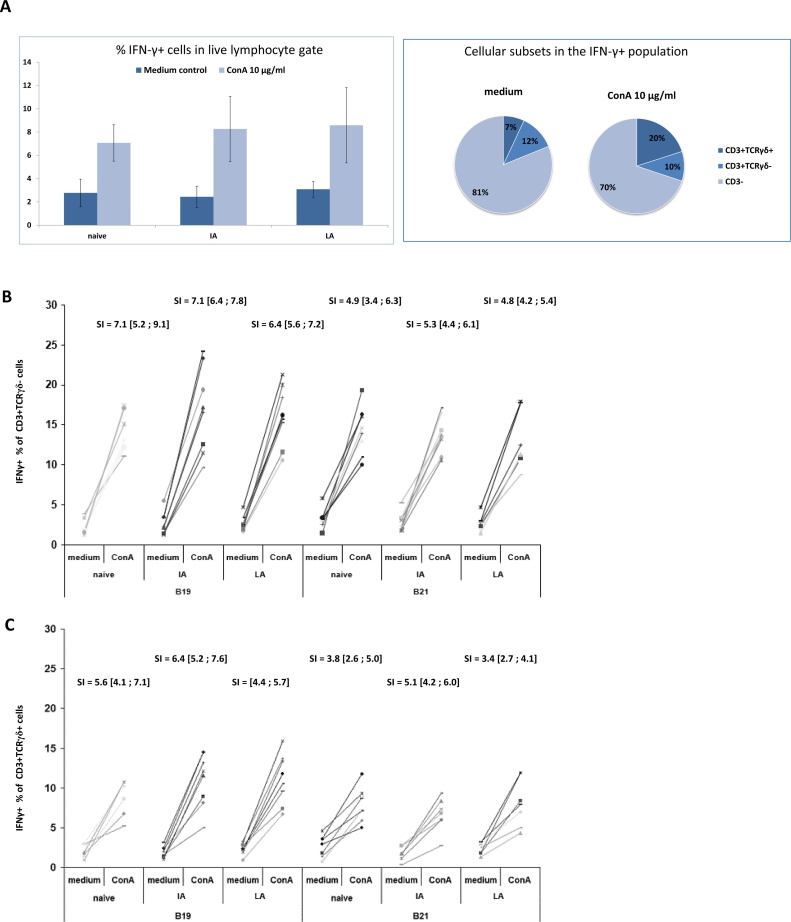
**The phenotype and frequencies of IFN-γ producing cells upon ConA stimulation.** A)The frequency of IFN-γ positive cells within the live lymphocyte gate (left) – mean values are shown (n = 16) +/- SD. The proportion of cells being CD3^-^, CD3^+^TCRγδ^-^ or CD3^+^TCRγδ^+^ in IFN-γ^+^ population (right). The percentages shown are mean values of all chickens in the experiment (n = 48). B) & C) Frequencies of IFN-γ^+^ cells in the CD3^+^TCRγδ^-^ population or the CD3^+^TCRγδ^+^ population of PBMC samples from naive and NDV immune animals either immunised with inactivated (IA) or live attenuated (LA) vaccine with and without stimulation with 10 μg/ml ConA. Data from individual chickens are shown with stimulation indexes (SI) indicated above including 95% confidence intervals in brackets.

ConA stimulation was performed on PBMC collected at five weeks PV2. The phenotype and proportion of live lymphocytes producing IFN-γ post ConA stimulation were assessed by ICS and flow cytometry (gating strategy shown in Fig. 3A and B). A large proportion of the IFN-γ producing cells were CD3^−^ (Fig. 3A). Only 19% of the IFN-γ producing cells observed in the medium controls and up to 33% in the stimulated samples were CD3^+^ by surface staining (data not shown). The IFN-γ producing CD3^+^ cell population comprised both TCRγδ^+^ and TCRγδ^−^, *i.e.* TCR αβ cells. No differences were observed between the groups in the total percentage of IFN-γ^+ ^cells in the live lymphocyte gate (Fig. 4A, left). Interestingly, ConA had little effect on the proportion of CD3^+^ TCRγδ^−^ IFN-γ producers which were 12% in medium controls compared to 10–11% in ConA-stimulated samples (Fig. 4A, right). In contrast, the proportion of CD3^+^ TCRγδ^+^ IFN-γ producers increased from 7% in medium controls to 20–22% in ConA-stimulated samples (Fig. 4A, right). No differences in MFI of the IFN-γ positive cells were observed (data not shown).

To assess if ConA induced IFN-γ production was affected by NDV vaccination and/or chicken MHC haplotype, stimulation indices (SI; IFN-γ^+^ proportion in stimulated samples/IFN-γ^+^ proportion in medium controls) were calculated for the CD3^+^ TCRγδ^−^ population (Fig. 4B) and the CD3^+^ TCRγδ^+^ population (Fig. 4C). However, no statistically significant differences were observed between MHC haplotypes or treatment groups from cells unstimulated or stimulated with ConA.

### 3.6 IFN-γ production in *ex vivo* NDV-activated PBMC

**Fig. 5:**
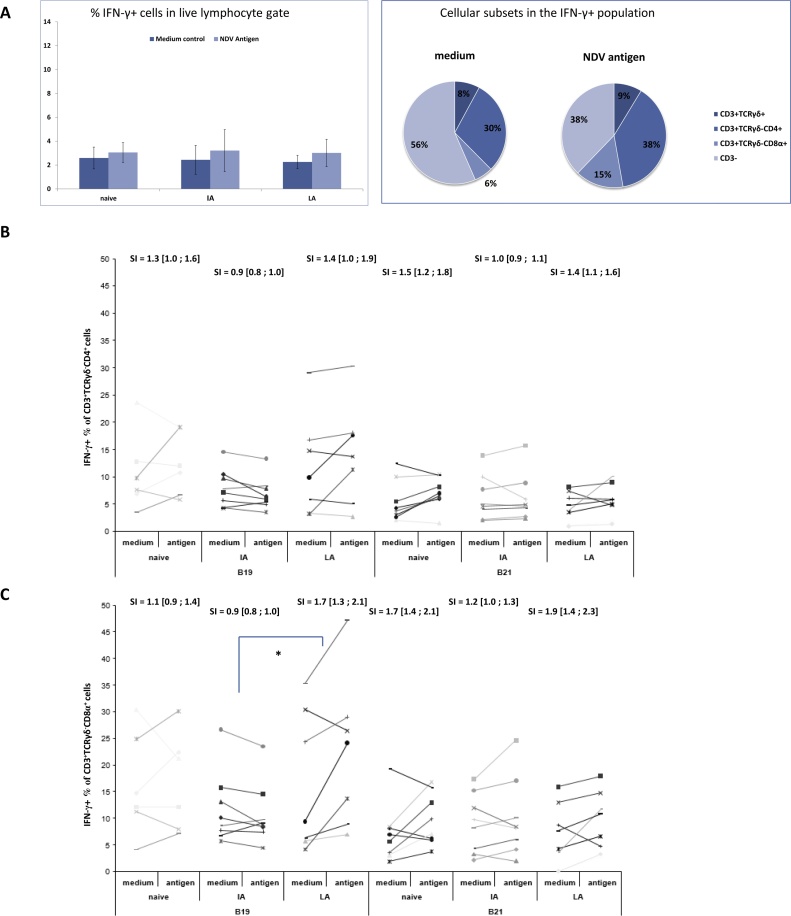
**The phenotype and frequencies of IFN-γ producing cells upon (NDV) antigen stimulation.** A) The frequency of IFN-γ positive cells within the live lymphocyte gate (left) – mean values are show (n = 16) +/- SD. The proportion of cells being CD3^+^TCRγδ^+^, CD3^+^TCRγδ^-^CD4^+^, CD3^+^TCRγδ^-^CD8α^+^ or CD3^-^ in the IFN-γ^+^ population (right). The percentages shown are mean values of all chickens in the experiment (n = 48) +/- SD. B) & C). Frequencies of IFNγ^+^ cells in the CD3^+^TCRγδ^-^CD4^+^ population or CD3^+^TCRγδ^-^CD8α^+^ population of PBMC samples from naive and NDV immune animals either immunised with inactivated (IA) or live attenuated (LA) vaccine, with and without stimulation with NDV antigen. Data from individual chickens are shown with stimulation indexes (SI) indicated above including 95% confidence intervals in brackets. Significant SI differences are indicated by * (P < 0.05).

NDV stimulation was performed on PBMC collected at 10 weeks PV2. The phenotype and proportion of live lymphocytes that produced IFN-γ upon stimulation were assessed by ICS and flow cytometry (gating strategy shown in Fig. 3A and C). During optimisation we concluded that staining benefitted from overnight rest before antigen stimulation which was not the case for ConA stimulation. The overnight rest, led to a larger proportion of IFN-γ producing CD3^+^ cells in the medium controls. In total, 44% of the cells were CD3^+^ in the medium controls compared to 66% in the antigen-stimulated samples (data not shown). No differences were observed between the groups in the total percentage of IFN-γ^+^ cells in the live lymphocyte gate (Fig. 5A, left). The CD3^+^ IFN-γ^+^ cell population comprised both TCRγδ^+^ and TCRγδ^−^ cells. Furthermore, the CD3^+^ TCRγδ^−^ population consisted of both CD4^+^ and CD8α^+^ cells. NDV antigen stimulation had little effect on the proportion of CD3^+^ TCRγδ^+^ IFN-γ^+^ cells which constituted 8% in medium controls and 9% in antigen-stimulated samples of the live lymphocytes (Fig. 5A, right). In contrast the proportion of CD3^+^ TCRγδ^−^ IFN-γ^+^ cells (*i.e.* the CD4^+^ and the CD8α^+^) increased from 36% in medium controls to 53–57% in antigen-stimulated samples (Fig. 5A, right). In all samples, the MFI of the IFN-γ positive cells were 10–20% higher in the CD3^+^TCRγδ^−^CD8α^+^ population as compared to the CD3^+^TCRγδ^−^CD4^+^ population (data not shown).

The effects of NDV vaccination and/or chicken MHC haplotype on NDV induced IFN-γ production were assessed in the TCRγδ^−^ population by calculating SI for the CD3^+^CD4^+^ and the CD3^+^CD8α^+^ population. In the CD3^+^TCRγδ^−^CD8α^+^ population of B19 chicken, SI of IFN-γ producers upon antigen stimulation was higher in the LA vaccinated group compared to the IA vaccinated group and the naïve controls (although the latter was not statistically significant; Fig. 5C). For the B21 chickens, SI for CD3^+^TCRγδ^−^ CD8α^+^IFN-γ^+^ population did not differ between treatment groups. Moreover, no significant differences between groups or MHC haplotypes were observed in SI of the CD3^+^TCRγδ^−^CD4^+^ population post NDV-stimulation (Fig. 5B).

## 4 Discussion

The present study aimed to validate currently available antibodies against chIFN-γ for use in a flow cytometry based ICS assay. We aimed to phenotype IFN-γ producing chicken lymphocytes in peripheral blood from NDV vaccinated chickens using *ex vivo* stimulation with polyclonal mitogen, ConA as well as specific NDV antigen. It was earlier shown that it is possible to assess IFN-γ production of splenic chicken T cells by ICS using the BD Cytofix/Cytoperm™ kit which was developed for mammalian cells (Ariaans et al., 2008, Ruiz-Hernandez et al., 2015). In the present study we have further optimised the ICS protocol and applied it to PBMC from a vaccine study where inbred MHC characterised chickens were immunised with live attenuated and inactivated Newcastle disease vaccines.

For initial testing of various antibodies, we found that CHO cells expressing recombinant chIFN-γ were very useful. Others have previously shown that BFA increases intracellular accumulation of cytokine (MFI) in the CHO transfectants without altering the percentage positivity of the cells (Entrican, 2002). However, in our experiments BFA increased both the percentage of positive cells as well as the MFI. Moreover, we observed differences in the ability of some antibodies to recognise recombinant chIFN-γ as compared to native chIFN-γ (data not shown), reflecting a general problem. Hence, it is essential, during the development of new chicken reagents to perform quality control ensuring the suitability of *e.g.* new cytokine antibodies for detection of native chicken cytokines.

As expected we observed a large difference in the humoral vaccine response between chickens immunised with the IA or the LA vaccine (Fig. 2), which is in accordance with our previous experiments using the inbred chicken lines (Dalgaard et al., 2010a). This is supported by work by others showing that, in general birds vaccinated with inactivated vaccines, tend to have higher humoral antibody levels although they do not develop a strong cell mediated response (Schijns et al., 2014, Dimitrov et al., 2017).

We were unable to detect vaccine-induced differences by analysing ConA activated PBMC in this study (Fig. 4B and C). This is in agreement with previous results obtained by chIFN-γ capture ELISA analyses of the supernatant of polyclonal-stimulated splenocytes in which no significant differences were observed between naive and NDV-vaccinated chickens (Lambrecht et al., 2004). Interestingly, not all IFN-γ producing cells were found to be surface CD3^+^ (Fig. 4A) suggesting IFN-γ is produced by other cell types. This is a well-known issue in mammalian research when using IFN-γ release assays. Several investigators have suggested that not only T cells produce IFN-γ following overnight stimulation with mitogen or antigen (Hagiwara et al., 1995, Vitale et al., 2000). Indeed, Desombere et al., 2004, Desombere et al., 2005 reported that overnight stimulation of human PBMC led to IFN-γ produced majorly by NK cells whereas after 5-days of stimulation most IFN-γ producing cells were T lymphocytes. However, in chickens, as opposed to mammals, the population of NK cells in blood constitutes only around 1% of peripheral lymphocytes (Neulen et al., 2015). The identity of the CD3 negative IFN-γ producing cells in the current study remains unknown at present. Due to well-known issues regarding changes in forward/side scatter profiles of PBMC after fixation, we cannot exclude the possibility that our lymphocyte gate contain monocytes and that they form part of the CD3 negative IFN-γ producing population. It is also possible that the CD3^−^ IFN-γ producing population contains thrombocytes. Avian thrombocytes are nucleated platelets forming a large part of peripheral blood (Seliger et al., 2012) and have been shown to have roles in inflammation and antimicrobial defence (Scott and Owens, 2008, Ferdous and Scott, 2015, Ferdous et al., 2016). However, it is controversial for how long thrombocytes survive in culture. Kaspers and Kaiser (2014) state that these cells die at 48–72 h. DaMatta et al. (1999) shows that about 70% of the thrombocytes dies by apoptosis by 24 h and 85% by 48 h. Certainly, thrombocyte cytokine gene expression was reported up to 18 h in culture (St Paul et al., 2012). In conclusion, further ICS studies are needed using specific thrombocyte and monocyte marker antibodies to define the observed CD3 negative population.

We observed an increase in the proportion of CD3^+^ IFN-γ producing cells after stimulation with ConA. However, while the proportion of IFN-γ producing CD3^+^TCRγδ^+^ cells doubled in these cultures, the proportion of IFN-γ producing CD3^+^TCRγδ^−^ (TCRαβ cells) was unaltered. It has been reported that chicken γδ T cells are poor cytokine producers and dependent on αβ T cells for their activation and proliferation (Kasahara et al., 1993). The present study showed, however, that 2–16% of the γδ T cells were able to produce IFN-γ upon ConA stimulation. In another experiment, we observed prominent expansion and blast transformation of TCRγδ^+^CD8β^+^ splenocytes upon *in vitro* ConA stimulation in combination with TCRγδ^−^CD8β^+^ cells in the cultures showing 10-fold higher responses (unpublished observation). Thus, it seems that chicken TCRγδ cell populations may be activated by mitogenic substances and depending on the trait monitored with or without corresponding responses in the TCRαβ cell population. In conclusion, methods that enumerate cytokine producing cells without determining their phenotype *e.g.* by ELISPOT methodology should be interpreted with caution if the results are to indicate solely classic T lymphocyte activity.

To address differences in the antigen-specific T cell responses, the IFN-γ induction protocol was changed to include an overnight rest before stimulation with NDV antigen. In mammals, PBMC or whole blood is widely used in recall assays as described in the current study. However, the antigen presentation potential of the PBMC samples were reported to be of importance in successful T cell activation *ex vivo* (Thiel et al., 2004). In general, exogenous antigens are easily processed by antigen-presenting cells and displayed as peptides of MHC-II molecules thus stimulating CD4^+^ T cell activation. In a study by Maecker et al., (2001), cross priming where peptides are displayed on MHC-I and presented to CD8^+^ T cells was also shown to occur. However, higher doses of whole protein were needed in order to observe stimulation efficiency comparable to that of synthetic peptides. Interestingly the ability to efficiently cross-prime was not found to be correlated to the number of dendritic cells in the donor’s blood but rather the number of monocytes (Maecker et al., 2001). In chickens we have also reported the importance of monocytes in PBMC samples in order to obtain efficient CD8^+^ T cell stimulation (Dalgaard et al., 2016). Further, studies are needed in order to pinpoint the exact mechanisms and peripheral blood cell types involved in antigen presentation in chicken *ex vivo* T cell assays.

In the current study, the frequency of IFN-γ producers in the CD3^+^TCRγδ^−^CD8α^+^ population upon NDV-specific stimulation was significantly higher in the group vaccinated with live attenuated NDV vaccine as compared to the group vaccinated with inactivated NDV (Fig. 5C). However, this was only observed for B19 chickens and not for B21 chickens. This inbred chicken line was earlier used in a comparable NDV vaccination study where antigen-specific T cell responses were assessed by CFSE staining and proliferation analyses (Dalgaard et al., 2010b). PBMC from chickens (of both MHC haplotypes) immunised with live attenuated vaccine showed a significantly higher proliferative capacity upon virus-specific recall stimulation than naive MHC-matched controls. However, no significant differences between the two haplotypes were reported (Dalgaard et al., 2010b). Nevertheless, the contrasting results may reflect that proliferating T cells do not necessarily produce IFN-γ and vice versa. This was previously suggested by Lambrecht et al. (2004) when no close correlation was found between the proliferative response to mitogens and the IFN-γ production in chicken splenocyte cultures. The observed antigen-specific IFN-γ production in CD3^+^TCRγδ^−^CD8^+^ cells of B19 chickens was only observed in the group vaccinated with the live attenuated vaccine and not in the group having received the inactivated vaccine. This was in good agreement with earlier reports demonstrating that live attenuated NDV vaccines induce a stronger CMI than inactivated NDV vaccines (Al-Garib et al., 2003, Zoth et al., 2008, Lambrecht et al., 2004, Rauw et al., 2009, Jayawardane and Spradbrow, 1995). However, the observation may not only reflect differences in the amount of circulating memory T cells induced by the vaccinations, but also differences in vaccine response kinetics and hence the possibility of identifying the NDV specific cells in peripheral blood. It should also be noted that recall stimulation using *e.g.* synthetic peptide pools or recombinant viral proteins may improve the methodology. The NDV antigen used in this study most likely, apart from viral protein/peptides, also contain unspecific stimuli, such as viral nucleic acids and other viral pathogen associated molecular patterns. Hence, the IFN-γ producing cells were not necessarily exclusively responding to NDV peptides presented by MHC. Interestingly, spontaneous IFN-γ production by unstimulated lymphocytes (medium controls) was earlier reported to be higher in samples from AIV infected chickens as compared to uninfected controls (Reemers et al., 2012). In general, we also observed large differences in the spontaneous IFN-γ production in medium controls between treatment groups, MHC type and sample time as previously reported in various species (Ariaans et al., 2009, Koets et al., 2006).

The study of chicken antigen-specific T cells is much hampered by the lack of available MHC multimer reagents, and thus knowledge of where and when to detect specific effector and memory T cells *in vivo* is scarce. The fact that the chickens *e.g.* lack draining lymph nodes as we know them from mammals further complicates a comparative approach. However, a few reports have established the presence of virus-specific memory T cells in both spleen and peripheral blood of chickens. Thus, Singh et al. (2010) reported assessment of *ex vivo* stimulated avian influenza virus specific peripheral T cells by their IFN-γ secretion (by NO inducing capacity in the HD11 cell line) and found cytokine producing capacity 3–9 weeks post a DNA vaccination. Likewise, Pei et al. (2003) reported the presence of Infectious Bronchitis virus (IBV) specific T cells in the spleen 3–6 weeks after infection by assessment of their protective potential by adoptive transfer. In the future, assessment of chicken memory T cell biology will be aided by development of new MHC multimer reagents and identification of T cell activation and memory cell markers for detailed phenotyping of antigen-specific cells. In addition functional assays for assessment of combinations of parameters such as proliferative capacity, degranulation potential/CTL activity and ability to produce several cytokines in addition to IFN-γ will be key to progress in the field.

In conclusion, we found that the ICS method could be useful for studying vaccine-induced T cell responses in the chicken but careful optimisation should be performed in each experimental setup. Although ConA increased the frequencies of IFN-γ^+^ cells, it is noteworthy that a large proportion of the IFN-γ producers were γδ T cells or even thrombocytes. Furthermore, the ICS method was used for assessment of NDV-specific T cell responses in a vaccination experiment and large variations between individuals were observed along with a non-specific background response in naive chickens. In order to further pursue antigen-specific responses, optimisation of the NDV antigen stimulation protocol must be done, *e.g.* by co-stimulation strategies or improved antigen presentation by APC enrichment. Furthermore, not only the magnitude but also the quality of a T cell response is important for protective immunity, as shown in mammalian studies (Seder et al., 2008). In the future, it will be necessary to address combinations of T cell functions in the chicken to identify valuable correlates of protection.

## Conflicts of interest

The authors declare no conflicts of interest.
