# Supplementary material for: Quantification and phenotypic characterisation of peripheral IFN-γ producing leucocytes in chickens vaccinated against Newcastle disease
Source: Vet Immunol Immunopathol. 2017 Dec;193-194:18–28. doi: 10.1016/j.vetimm.2017.10.001 (PMC5697524; doi:10.1016/j.vetimm.2017.10.001)
Supplement: Supplementary file 1 [file mmc1.pdf]

Supplementary Figure 1

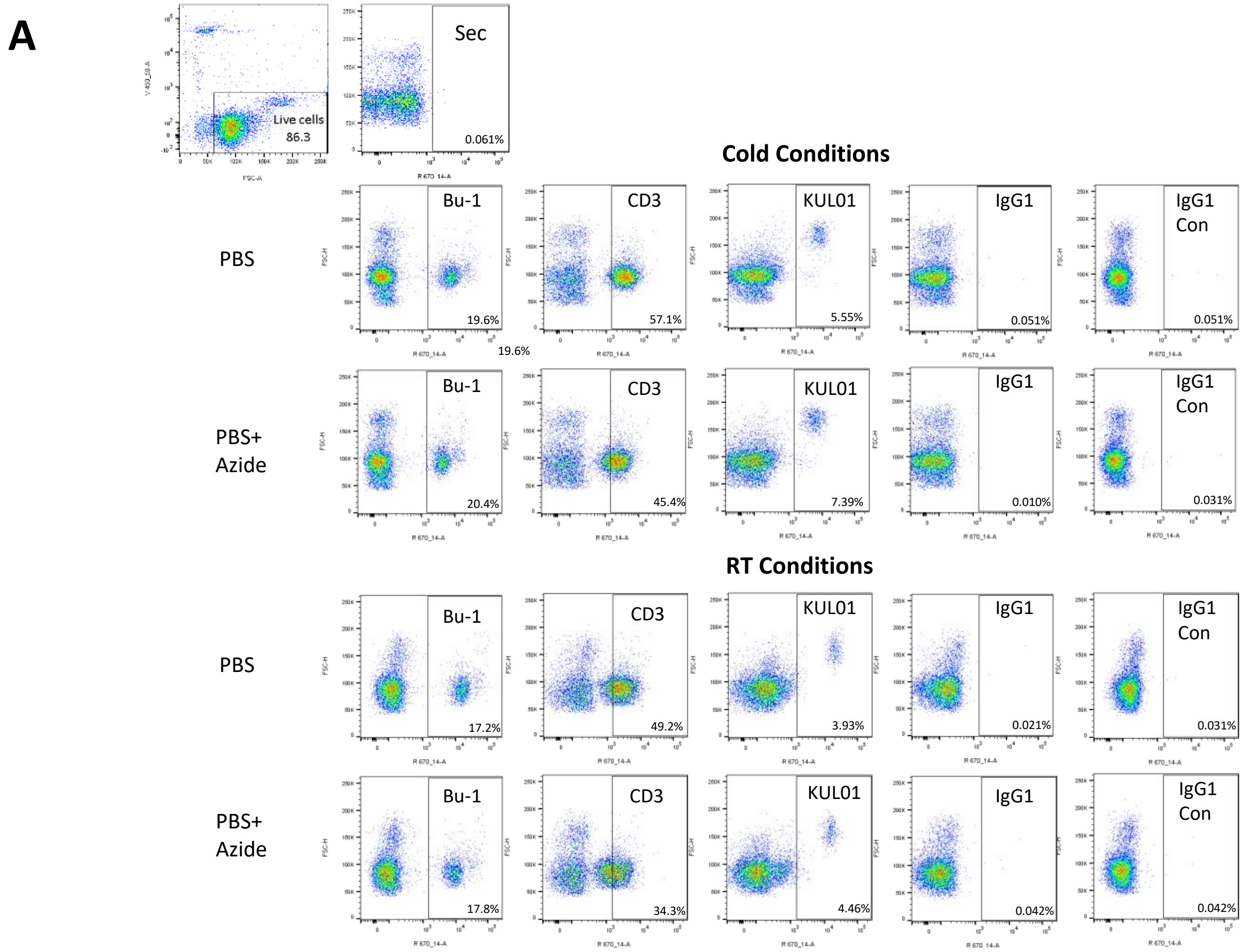

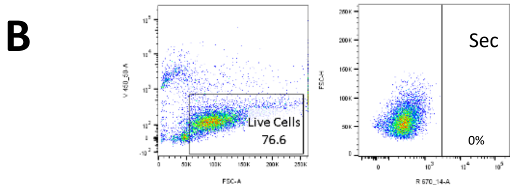

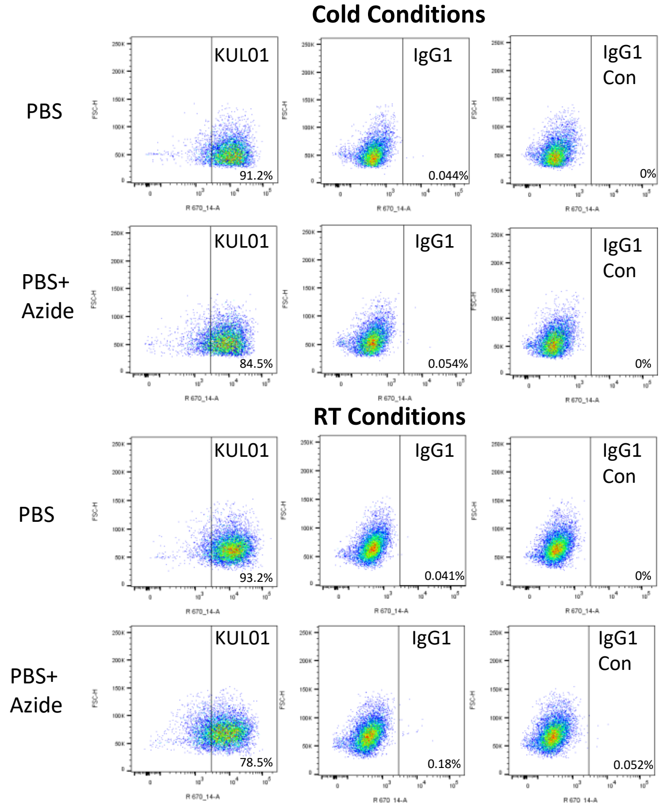

Supplementary Figure 1. Analysis of mouse IgG staining and endocytosis under various conditions by flow cytometry.
Live cells were analysed by Sytox blue exclusion A) PBMC and B) BMDC. PBMC were stained with a reference antibody, Bu1-Alexa Fluor® 647, unconjugated CD3, and all cells were stained for KUL01 followed by goat anti-mouse Alexa Fluor 647 IgG1. Mouse IgG uptake/binding was analysed using mouse IgG1 conjugated to Alexa Fluor® 647 (IgG1 Con) and unconjugated mouse IgG1 (IgG1) followed by goat anti-mouse Alexa Fluor® 647 IgG1. Stainings were carried out on ice (cold) or RT conditions in the presence of PBS or PBS/Azide. Gates were applied to live cells using secondary staining controls (Sec). Data is representative of three technical replicates.
