# Supplementary material for: Quantification and phenotypic characterisation of peripheral IFN-γ producing leucocytes in chickens vaccinated against Newcastle disease
Source: Vet Immunol Immunopathol. 2017 Dec;193-194:18–28. doi: 10.1016/j.vetimm.2017.10.001 (PMC5697524; doi:10.1016/j.vetimm.2017.10.001)
Supplement: Supplementary file 2 [file mmc2.pdf]

Supplementary Figure 2

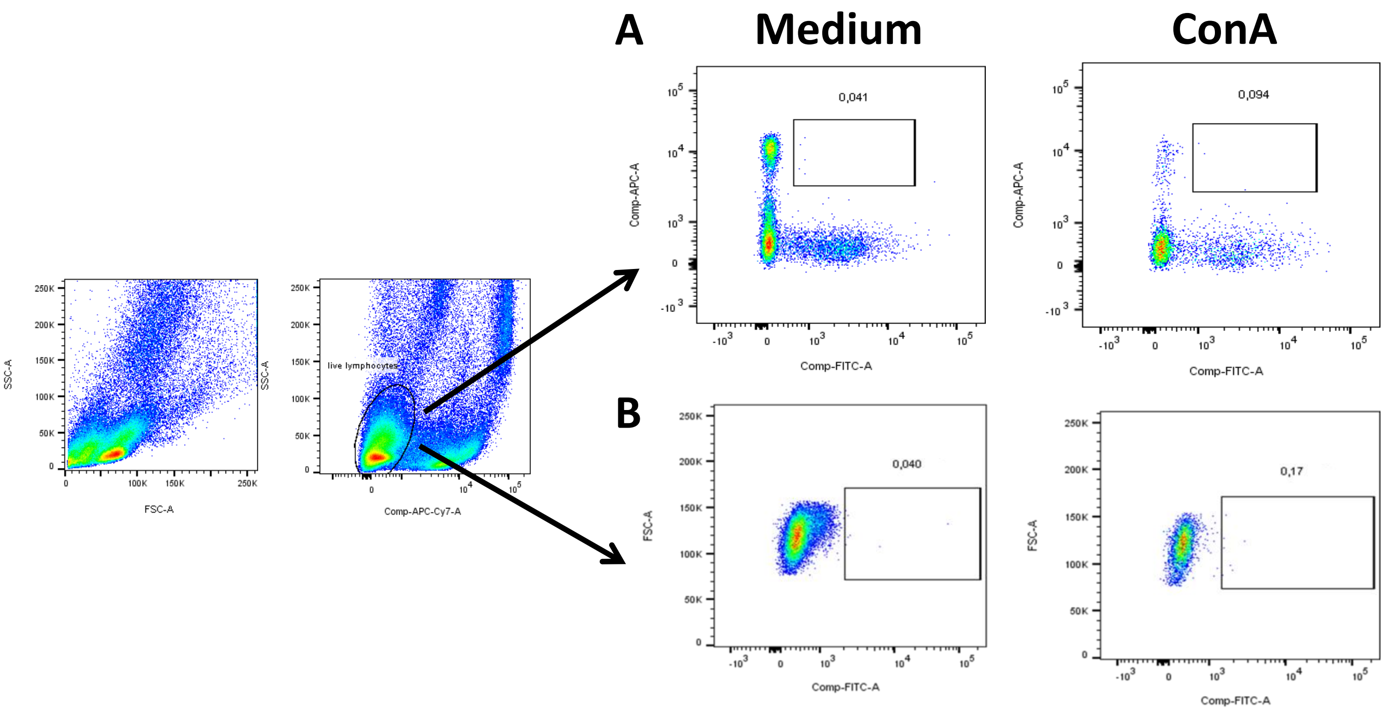

Supplementary Figure 2. ICS staining controls.
Intracellular staining of PBMC, left panel medium controls, right panel PBMC stimulated with ConA (5 µg/ml ConA, 18 hours incubation). Shown only cells in live lymphocyte gate (live cell discrimination by Near-IR live/dead cells stain). A) PBMC double stained mouse anti-chicken CD8α-Cy5 antibody in combination with irrelevant rabbit polyclonal antibody (Rabbit anti-chicken IgG FITC). B) PBMC stained with secondary goat anti rabbit IgG FITC only.
