# Supplementary material for: Quantification and phenotypic characterisation of peripheral IFN-γ producing leucocytes in chickens vaccinated against Newcastle disease
Source: Vet Immunol Immunopathol. 2017 Dec;193-194:18–28. doi: 10.1016/j.vetimm.2017.10.001 (PMC5697524; doi:10.1016/j.vetimm.2017.10.001)
Supplement: Supplementary file 3 [file mmc3.pdf]

Supplementary Table 1. ICS variables evaluated during optimisation. PBMC from naive (n=4) and NDV immune (n=4) chickens were tested in triplicates.

Variable	*Conditions tested (bestchoice in bold)*	*Reason for choice*
Mitogen added at	**Day 0**<br>Day 1	PBMC rested overnight in the culture plates responded poorly to ConA stimulation.
Antigen added at	Day 0<br>**Day 1**	PBMC rested overnight in the culture plates before stimulation with NDV antigen showed an increase in the frequency of IFN-γ producers as compared to stimulation at day 0 directly after PBMC isolation.
Culture medium	R10<br>**X-VIVO15**<br>**CTL**	Stimulation of PBMC from naive chickens with ConA (10 μg/ml) showed a slightly higher frequency of IFN-γ producers (but not significant) when grown in serum free media as compared to R10.
Detachment of activated cells from plastic surface	**+ 2mM EDTA**<br>- 2mM EDTA	After overnight stimulation with 10 μg/ml ConA in the presence of 5 μg/ml BFA it was shown that treatment with 2 mM EDTA increased the frequencies of IFN-γ producing cells.
Stimulation time	6 h<br>**Overnight**	Stimulation with 10 μg/ml ConA in the presence of 10 μg/ml BFA for 6 h showed a lower IFN-γ response compared to 10 μg/ml ConA in the presence of 5 μg/ml BFA overnight. More dead cells were present after overnight stimulation.
Viability marker	**+ ViD**<br>- ViD	Dead cells led to false positive stainings. The fixable viability marker allowed us to gate out this population.

Please note that conditions stated in this table are not universal. Other conditions may apply for different antigen candidates (e.g. viral lysate, recombinant viral proteins, overlapping synthetic peptides).
